# Faults of Medical Writers

**Published:** 1854-01

**Authors:** 


### ECLECTIC DEPARTMENT,

### AND SPIRIT OF THE MEDICAL PERIODICAL PRESS

We have read the following extract with pleasure. It is a defense of the purity of our mother tongue, a vindication of its strength and capacity for expression. No class of writers are more open to criticism on the use of bastard and unlineal words, than those who fructify the medical profession. A man would need the gift of tongues to comprehend the manifold words which may be found in medical literature, but not in any lexicon. While we not unfrequently abandon in despair the perusal of some of these stilted essays, written in Carylese run mad, we turn with pleasure to the straight-forward, manly and *English* style of others, who write because they have something to say; something which they wish to be understood; and whose "every word weighs twelve pounds." S. B. H.

Faults of Medical Writers.—In the discourse by Dr. Samuel Jackson, before the Philadelphia County Medical Society, at its last annual meeting, we find the following remarks on a subject which deserves the attention of the profession generally—especially those who are in the habit, as all should be, of writing occasionally for the press:—

"Let the young doctor do his very utmost in acquiring a habit of writing with *perspicuity, propriety and precision.* Let him seek no other ornament, for medical language is, like Thompson's loveliness, when 'unadorned, adorned the most.' No merit will make amends for the want of perspicuity. I can show whole paragraphs in our American books which have no meaning whatever, being similar in this respect to those verbose letters that Queen Elizabeth used to write when she had pre-determined to say nothing. Medical diction ought to use as few words as possible, thus going the shortest way to the end of a thought. An English writer on morbid poisons, wishing to describe the daily progress of the variolous pustule, uses the following verbosity: 'You receive from a long distance, from Dublin or from Edinburgh, a lancet, on the point of which there is a little dry animal matter. This lancet has pricked the pustule of a patient suffering with smallpox, and the contents of the pustule have been suffered to dry on the lancet. Now with this lancet you make a single puncture in the arm of a healthy person, not previously defended by vaccination or otherwise, and what results?

Now suppose this author, Dr. Simon, had wished to describe also, the effect of a rattlesnake's bite; he might have begun thus: You receive from a long distance, from Utah or California, a rattlesnake, which Linnæus calls *crotalus,* it may be the species *horridus* or *durisus;* this dreadful animal has a sacculus of poison at the root of each fang, and when he bites, these sacculi pour forth their deadly contents along a groove in each fang. Now you permit this animal to bite a horse, for an experiment, or perhaps it bites one of you, and what results? In this multiplication of useless verbiage, a great amount of time is wasted without any compensation.

In a celebrated medical journal, we have this circuitous way of saying that a certain medicine was probably useful in rheumatism; the disease was cured in eleven days; "and lemon juice, if it was not the principal remedy, certainly exerted an important influence toward the production of that end." What think you, gentlemen, of *producing* or *leading forward* an end or a cure? One might suppose that the writer was a cobbler, and that he was talking about the *producing* or the *pulling forward* of his waxed-end. And then he has lemon-juice *making an exertion, and exerting an influence.*

Why should a writer say, "I had recourse to a medicine," if he had not previously used it in the same disease? This word means a running backward. The simple English word to *give,* is often supplanted by the Latin word to *exhibit;* that is, to make a show of the medicine. A shopkeeper *exhibits* his goods, a physician *gives* or *orders* his medicine. Celsus took nearly all his ideas from the Greeks, but he did not copy their words. I

believe he never uses the word *exhibere*, but *dare et uti*. Sometimes he says *adhibere*, but this does not mean *to make a show;* moreover, it is pure Latin. His own language was sufficient for him, except in the mere naming of diseases; and hence one reason that his style and manner are universally approved.

It is of no little importance that our young author should not practise the coining of words. A new idea may require a new word, but old ideas will always be most intelligibly introduced by known terms; hence the great English lexicographer, whose head might well be fancied as swarming with words, introduced only four in all his writings. His rule was, "to admit only such as may supply real deficiencies, such as are readily adopted by the genius of our tongue, and incorporate easily with our native idiom." If a little license be granted, how will you define its limits? How will you definitely measure the old vulgar phrase *too much?* A little liberty will prove like moderate drinking, and lead to intemperance. If every writer of the present time should coin words at his pleasure, and the next generation should adopt them and add to them, what odious gibberish would then fill the air! It is told of Sir John Mandeville, that, when far in northern Asia, with his retinue, their words were all frozen before they could be heard, and that, on coming south, they were suddenly thawed, and filled the air with their liberated voices. I can hardly credit this fact, as the amiable author does not relate it himself, and yet something similar may happen to the jargon of the present generation; while confined to books it may pass without much notice, but our successors may find the accumulated vocabulary to become a clattering of unmeaning voices, the mere echoes of our vanity, and as unintelligible as Sir John's thawed vocables.

In the Transactions of the American Medical Association you may find some animating specimens of these important additions to our deficient language. *Numerism, socialism, sensationalism, subjectivity, progressionist, therapeutication, truths eliminated, annexes of the heart.* A writer in vol. iv., p. 59, calls impressions "*intuitively-felt relations*," and then inquires, "Are not all the felt relations based on immediacy and intuition, and not on representational and transmitted impressions." Truly, if men in high places continue to pour forth such floods of impurity, men in low places may well complain; hence I have ventured to notice the subject; it pertains to *self-education*, which is our present topic.—*Boston Medical and Surgical Journal.*

